# Effect of Pore Defects on Uniaxial Mechanical Properties of Bulk Hexagonal Hydroxyapatite: A Molecular Dynamics Study

**DOI:** 10.3390/ijms24021535

**Published:** 2023-01-12

**Authors:** Alexander D. Snyder, Iman Salehinia

**Affiliations:** 1 Department of Mechanical and Aerospace Engineering, North Carolina State University, Raleigh, NC 27695, USA; 2 Department of Mechanical Engineering, Northern Illinois University, DeKalb, IL 60115, USA

**Keywords:** molecular dynamics, hydroxyapatite, defects

## Abstract

Hydroxyapatite (HAP) is a calcium apatite bioceramic used in various naturally-derived and synthetic forms for bone repair and regeneration. While useful for the regrowth of osseus tissue, the poor load-bearing capacity of this material relative to other biomaterials is worsened by the propensity for pore formation during the synthetic processing of scaffolds, blocks, and granules. Here we use molecular dynamics (MD) simulations to improve the current understanding of the defect-altered uniaxial mechanical response in hexagonal HAP single crystals relative to defect-free structures. The inclusion of a central spherical pore within a repeated lattice was found to reduce both the failure stress and failure strain in uniaxial tension and compression, with up to a 30% reduction in maximum stress at the point of failure compared to a perfect crystalline structure observed when a 30 Å diameter pore was included. The Z axis ([0 0 0 1] crystalline direction) was found to be the least susceptible to pore defects in tension but the most sensitive to pore inclusion in compression. The deformation mechanisms are discussed to explain the observed mechanical responses, for which charge imbalances and geometric stress concentration factor effects caused by pore inclusion play a significant role.

## 1. Introduction

Human bone possesses remarkable mechanical properties that are both anisotropic and heterogeneous in nature, owing to the hierarchical structuring of both cortical and cancellous tissue regions [1]. At the smallest level, nanostructured (<1 μm) arrangements of collagen and hydroxyapatite (HAP) platelets and the sub-nanostructured molecular makeup of these components govern the mechanical response via deformation mechanisms spanning characteristic scales from nanometers to microns and involving multiple such arrays [2]. Skeletal structures are routinely subjected to complex loading involving tensile, compressive, and shear loading with flexible collagen fibrils acting as the matrix phase and transferring the load to the much stiffer HAP platelets via shear [3]. The chemical properties of HAP platelets are dependent on the base composition, which can be affected by local solvation in water and exposure to varying ionic and molecular species within osseous tissue [4]. HAP commonly forms either monoclinic or hexagonal lattice arrays, with the chemical formula Ca${}_{10}$(PO${}_{4}$)${}_{6}$(OH)${}_{2}$ consistent in both natural and synthetic forms. Synthetic forms more typically comprise a hexagonal lattice structure, and stoichiometric ratios are often readily preserved. Owing to the nanostructure-dependent osteoconductive and osteoinductive properties of HAP, synthetic forms are commonly used in resorbable bone tissue scaffolding and as a bioactive coating for bioinert materials [5,6,7,8,9,10,11,12]. Such applications demand well-controlled production of bulk HAP structures, for which mechanical properties are not well understood in application from a multiscale perspective [13]. This is compounded by the fact that synthetic HAP crystals typically possess common microscale defects such as pores, which can alter local mechanical behavior and failure inception. Significant experimental efforts have been conducted to understand the constitutive properties and failure mechanisms of synthetic HAP single crystals via nanoindentation, revealing size dependencies of dislocation pile-up and plasticity behavior as well as crystallographic anisotropy in hardness, work hardening response, elastic modulus, yield stress, and fracture toughness. [14,15,16]. However, physical testing lacks the ability to uncover the nanoscale deformation mechanisms ultimately responsible for such behavior—a deficiency that can be effectively mitigated via the use of well-developed simulation techniques such as molecular dynamics (MD). MD has been used to understand complex mechanical phenomena in HAP single crystals, including fracture confinement [17], and the temperature and strain-rate-dependent increase in anisotropic fracture toughness and elastic modulus of HAP [18]. When simulating larger systems, such as arrays of collagen fibrils reinforced with HAP platelets or mineralized with HAP, increasing platelet and/or mineralization content was correlated with the increased load-bearing response of the system [19,20]. MD has also been used to show that bulk HAP possesses tensile and compressive anisotropy, with the mechanical response governed by underlying nanoscale deformation mechanisms in the covalently and ionically bonded crystalline structure [21]. To date, however, computational studies of the mechanical response of bulk HAP possessing common manufacturing defects such as pores have not been performed—a knowledge gap that could properly inform future biofunctional HAP structures if addressed. In this work, we build upon our previous understanding of HAP mechanical properties by using molecular dynamics to assess the effect of spherical pore inclusion on the constitutive properties and failure stress of HAP bulk crystals subjected to uniaxial tensile and compressive loading. Spherical pores with diameters of 7.5, 10, 15, and 30 Å were placed centrally within a periodic HAP single crystal, and the failure behavior observed was comparable to a defect-free crystal. It was found that Z-axis (i.e., [0 0 0 1] crystalline direction) tensile behavior is the least sensitive to pore inclusion, while Z compression is the most sensitive.

## 2. Results and Discussion

In this study, we investigated the effect of a spherical pore in the center of the monocrystalline HAP model on its mechanical behavior under uniaxial loading. It was seen that the inclusion of a pore weakened the HAP crystal in all cases. This may be attributed to stress concentration at the pore edges as well as the creation of electrostatic imbalances in the structure depending on the bonds influenced by the pore formation. Observed stress concentration at the pore edges varies based on loading type. The pore was adjusted to have a diameter of 7.5, 10, 15, and 30 Å.

### 2.1. Uniaxial Tensile Behavior

**Figure 1:**
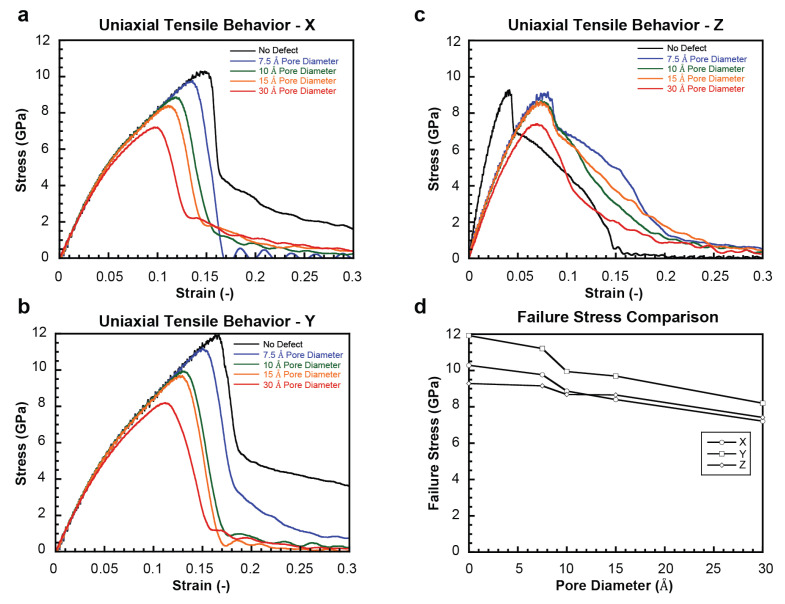
Effect of pore inclusion on HAP tensile mechanical response. (**a**–**c**) X, Y, and Z direction stress–strain curves for monocrystalline HAP with and without pores of different sizes, respectively. (**d**) Measured failure stress for each loading direction as a function of pore size.

Figure 1a–c show stress–strain plots for the pristine model and those with a central pore under uniaxial tension in the X, Y, and Z directions, respectively (see Figure 1). The typical behavior showing that stress increases to a maximum value before a significant drop indicates the inception of global failure in the crystal following this peak. The elastic region encompasses two sub-regions of differing slopes prior to the inception of material failure. For a pristine HAP crystal, it was shown that the failure of ionic bond groups was the dominant failure mechanism under tensile loading, i.e., Ca(I)-O(I), Ca(I)-O(III), Ca(II)-OH, and Ca(II)-O(I) bonds in X loading, Ca(I)-O(I), Ca(I)-O(III), Ca(II)-O(I), and Ca(II)-O(II) bonds in Y loading, and Ca(I)-O(II) and Ca(II)-O(III) variants in Z loading [21]. The reader is referred to our prior work for a more comprehensive overview of those ionic bonds and their contribution to the failure [21].

Figure 1d shows the variation of the measured stresses at the inception of failure with the pore size for each loading case. The tensile constitutive response is least affected by pore inclusion in the Z direction. This is likely due to the fact that the pristine nanoscale deformation mechanisms in this direction are largely comprised of extension and breakage of Ca(I)-O(II) and Ca(II)-O(III) bonds, which are distributed widely in the crystalline structure and interact through multiple X-Y atomic planes when repeated periodically. This is in contrast to X and Y tensile deformations, which are governed by the much greater (and more spatially concentrated) bonds that possess high X-Y planar character (i.e., most of the bond species on the Ca(I) and Ca(II) polyhedra). Thus, pore inclusion and increasing pore size have a greater effect in these directions as a large number of participating bonded groups can be eliminated within the pore diameter chosen [21]. Figure 1d shows that as the pore widens, convergence occurs between X and Z. This may be explained by the fact that the widening of the pore leads to the inclusion of Ca(I) atoms, which bond to surrounding oxygens such that the majority of Ca(I) bonds have a significant Z orientation.

**Figure 2:**
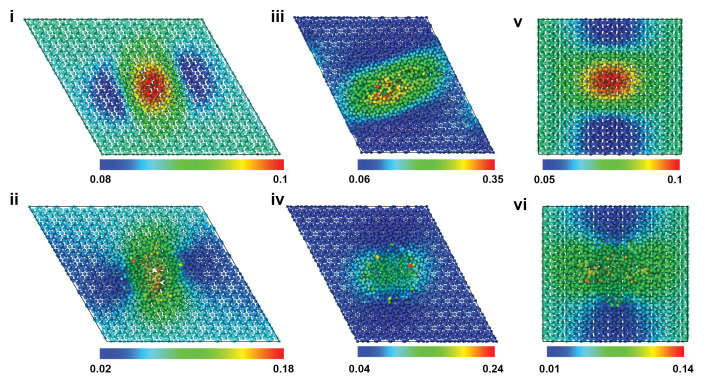
Atomic snapshots of HAP models at the inception of tensile failure for 7.5 Å (top row) and 30 Å (bottom row) pores, for X (**i**,**ii**), Y (**iii**,**iv**), and Z (**v**,**vi**) loading. Atoms are colored according to the von Mises strain. X and Y loading images are viewed in the X-Y plane, whereas the Z loading image is in the Y-Z plane.

Figure 2 shows the atomic snapshots of the HAP models at the inception of tensile failure for 7.5 Å (top row) and 30 Å (bottom row) pores, for X, Y, and Z loading directions. Atoms are colored according to the von Mises shear strain, which was calculated by comparing the atomic configuration of the models at the inception of failure to the ones before the tensile loading started [22]. The deformation/strain is highly localized around the pores, and the inception of failure happens from the pore and progresses outward. Similar strain values are seen for the different pore sizes. The deformation mechanisms and the range of shear strains for models with other pore sizes, i.e., 10 Å, 15 Å pores, were similar to the ones shown in this figure.

**Figure 3:**
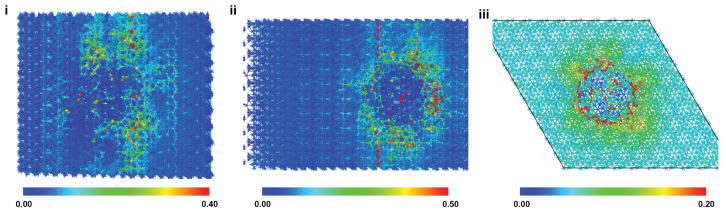
Atomic snapshots of HAP models containing 30 Å pores at the inception of tensile failure for X (**i**), Y (**ii**), and Z (**iii**) loading. Atoms are colored according to the atomic shear strain. X and Y loading images are viewed in the X-Y plane, whereas the Z loading image is in the Y-Z plane.

Further investigation of tensile deformation behavior in each direction was performed at the largest included pore size (30 Å) in an effort to ascertain if pristine crystal deformation mechanisms still governed the observed constitutive response. Figure 3 shows the contours of atomic shear strain through a half-section of the porous crystal in each loading direction at the respective point(s) of failure. Noticeable strain concentration is observed at the circumferential edges of the pore and not associated with any particular bonding type, indicative of Coulombic charge imbalances and geometric stress concentration factors playing a large role in mechanical response as opposed to specific bond deformations. This circumferential strain concentration is a common phenomenon even at low, i.e., elastic, deformations—indicating that unconstrained atomic motion at the edges of the pore due to “hanging” bonds is present.

### 2.2. Uniaxial Compressive Behavior

**Figure 4:**
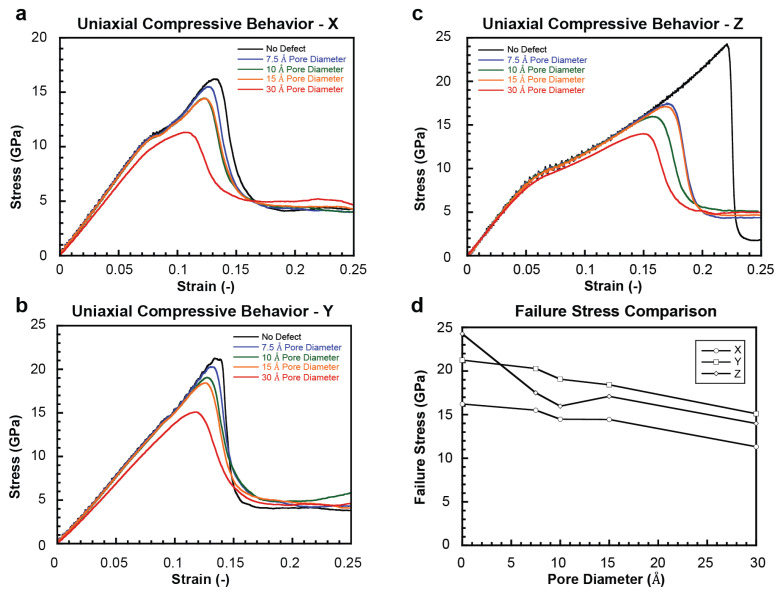
Effect of pore inclusion on HAP compressive mechanical response. (**a**–**c**) X, Y, and Z direction stress–strain curves for monocrystalline HAP with and without pores, respectively. (**d**) Measured failure stress for each loading direction as a function of pore size.

Figure 4a–c show stress–strain plots for the pristine model and those with a central pore under uniaxial compression in the X, Y, and Z directions, respectively. The overall behavior mirrors those in uniaxial tension, where the stress increases until the peak point and then drops due to the inception of failure. Snyder and Salehinia [21] showed that the Coulombic repulsion between ionic groups of similar charge was the dominant failure mechanism of pristine HAP crystals under compressive loading. The reader is referred to our prior work for a more comprehensive overview of the dominant deformation mechanisms for each loading direction [21].

Figure 4d shows the variation of measured stresses at the inception of failure with pore size for each loading case. In contrast to the uniaxial tension, the Z direction is the most sensitive to the pore size. The initial deletion of OH groups and surrounding Ca(II) sites causes uneven lattice strain as the remaining unbonded atoms Coulombically attract to nearby atoms of opposite charge. In this way, the structure and initial orientation of phosphate groups in the pore zone may be altered, leading to earlier instances of Coulombic repulsion between neighboring groups. The regaining of strength for the 15 Å pore diameter vs. 10 Å supports this conclusion—i.e., a larger pore may not necessarily eliminate a far greater number of bonded groups, meaning that pore size and induced charge imbalances are not necessarily directly related beneath certain size scales. Instead, groups with a pre-existing strain imbalance driving early failure at 10 Å could be eliminated, resulting in the elimination of the weakest point in the structure.

**Figure 5:**
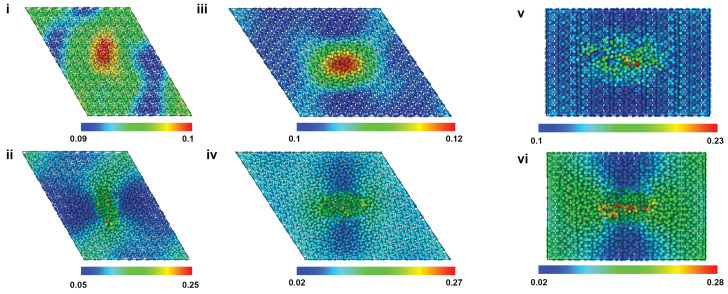
Atomic snapshots of HAP models at the inception of compressive failure for 7.5 Å (top row) and 30 Å (bottom row) pores, for X (**i**,**ii**), Y (**iii**,**iv**), and Z (**v**,**vi**) loading. Atoms are colored according to the von Mises shear strain. The view of the snapshots for X and Y loading is the X-Y plane, and it is the Y-Z plane for the Z loading.

**Figure 6:**
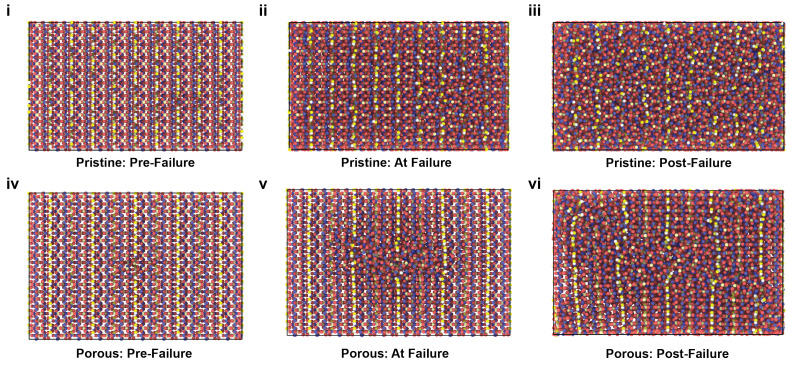
Atomic snapshots of the pristine HAP model (**i**–**iii**) and of the HAP model having a 7.5 Å pore (**iv**–**vi**) at various time-steps, i.e., before failure, at the inception of failure, and after failure under compressive loading in the Z direction. The view is in the Y-Z plane.

Figure 5 shows the atomic snapshots of HAP models at the inception of compressive failure for 7.5 Å (top row) and 30 Å (bottom row) pores, for the X, Y, and Z loading directions. Atoms are colored according to the von Mises shear strain. The snapshots show that the small pore was closed up before the inception of failure for all the loading directions. Snapshots of the models with the small pore, when compressed in X and Y directions, show a small difference between the lower and upper range of the strain, signaling a relatively uniform distribution of strain in the material. However, the strain range for the model with the small pore under compression in the Z direction shows significant localization of strain around the pore location. Snyder and Salehinia [21] linked the sudden and global failure of a pristine HAP crystal under the compressive loading in the Z direction to the continued flattening of oxygen groups between planes of phosphorus atoms. Even a small pore at the center of the HAP crystal disturbs the oxygen groups, resulting in localized failure of the crystal. To further clarify this observation, Figure 6 shows the atomic snapshots of the pristine HAP and the HAP with the small pore at various time-steps, i.e., before the failure, at the inception of failure, and after the failure. The inception of failure for the pristine crystal is global as the entire crystal fails at once; however, for the model with the pore, the failure starts from the pore and propagates into the crystal leading to global failure.

**Figure 7:**
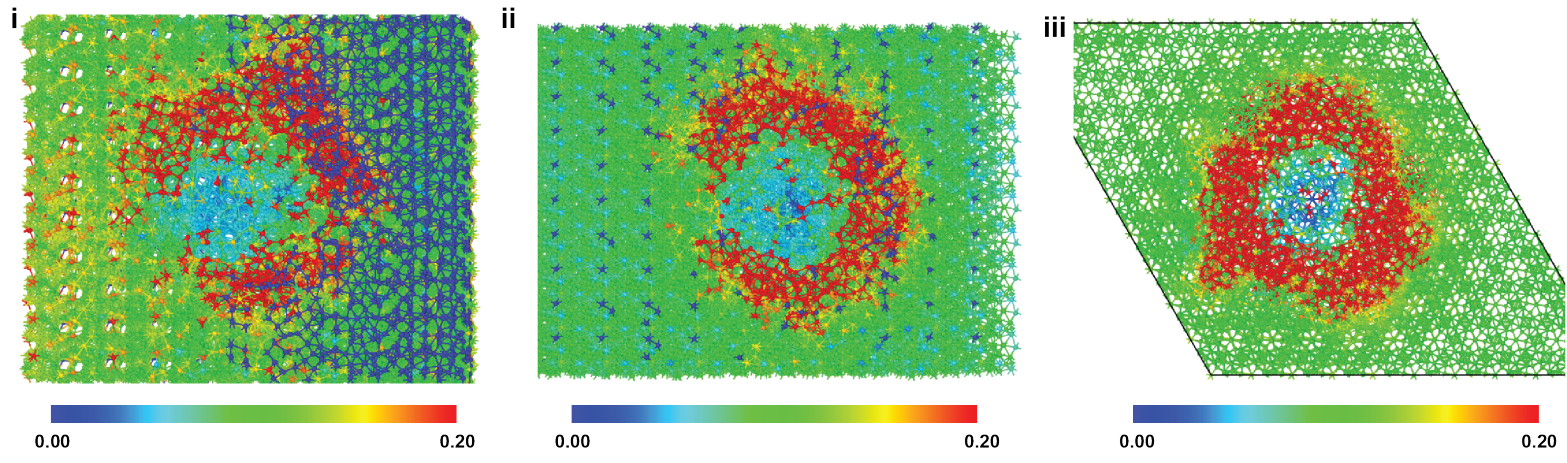
Atomic snapshots of HAP models containing 30 Å pores at the inception of compressive failure for X (**i**), Y (**ii**), and Z (**iii**) loading. Atoms are colored according to the atomic shear strain. X and Y loading images are viewed in the X-Y plane, whereas the Z loading image is in the Y-Z plane.

Similar to the tension results, noticeable strain concentration is observed at the circumferential edges of the pore in compressive loading and is not associated with any particular bonding type. This circumferential shear strain is persistent at all strain levels and is shown at the point of failure for each loading direction in Figure 7. While failure in pore-containing crystals has not been successfully linked to the sequential failure of specific bonded groups as for pristine HAP crystals, this does not disqualify the effect of pore-related lattice strain induced by the elimination of certain chemical groups and induced charge imbalances—factors worth decoupling and investigating further in future efforts.

## 3. Materials and Methods

The molecular dynamics studies shown in this work were performed using LAMMPS [23] with the CVFF-Interface Force Field interatomic potential, which has successfully reproduced the physical and chemical properties of hydroxyapatite compared to experiments in multiple studies [24,25,26,27,28]. The formulation of the CVFF-Interface Force Field interatomic potential is

$$\begin{array}{c} E_{total}=\Sigma_{bonds}K_{r,ij}{\left(r_{ij}-r_{0,ij}\right)}^{2}+\Sigma_{angles}K_{\Theta ,ijk}{\left(\Theta_{ijk}-\Theta_{0,ijk}\right)}^{2}+\Sigma_{ifnonbonded}\frac{q_{i}q_{j}}{4\pi \epsilon_{0}r_{ij}}+\\ \Sigma_{ifnonbonded}\epsilon_{ij}\left[{\left(\frac{\sigma_{ij}}{r_{ij}}\right)}^{12}-2{\left(\frac{\sigma_{ij}}{r_{ij}}\right)}^{6}\right] \end{array}$$

in which the primary summation is a harmonic bond stretching potential, the secondary summation is a harmonic bond angular potential, the tertiary summation is a non-bonded Coulombic interaction potential, and the quaternary summation is a 12-6 Lennard–Jones non-bonded interaction potential. Variables used in the formulation are defined as follows: $K_{r,ij}$ is the harmonic bond linear stretching stiffness, $r_{ij}$ is the center-to-center distance between two particles *i* and *j*, $r_{0,ij}$ is the initial length of a harmonic bond, $K_{\Theta ,ijk}$ is the harmonic bond angular stretching stiffness, $\Theta_{ijk}$ is the angle between three particles *i*, *j*, and *k*, $\Theta_{0,ijk}$ is the initial angle between three particles *i*, *j*, and *k*, $q_{i}$ is the charge of particle *i*, $q_{j}$ is the charge of particle *j*, $\epsilon_{0}$ is the permittivity of a vacuum, $\epsilon_{ij}$ is the Van Der Waals energy well depth, and $\sigma_{ij}$ is the Van der Waals radius of two non-bonded atoms *i* and *j*.

**Figure 8:**
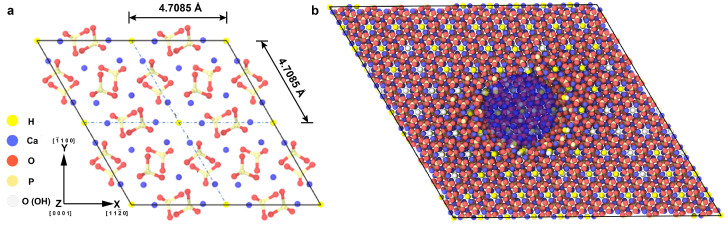
Simulated HAP domain. (**a**) The 44-atom HAP unit cell used as the base for replication. (**b**) Sectioned 44,000 atom HAP domain showing a 30 Å diameter pore at the center (shaded blue for clarity).

The HAP unit cells used in this work were constructed using Material Studio 4.0, with the base chemical formula of Ca${}_{10}$(PO${}_{4}$)${}_{6}$(OH)${}_{2}$ used in the construction of each 44-atom hexagonal lattice unit cell (Figure 8a). Replication of the unit cell was performed 10 times in the X, Y, and Z directions to obtain a size-insensitive simulation domain of 44,000 atoms. Bulk behavior was approximated via the application of periodicity in all directions. Defects were introduced into the system in the form of centrally placed spherical pores of 7.5, 10, 15, and 30 Å diameter (Figure 8b). Mechanical testing simulations were performed in tension and compression for the X, Y, and Z directions at a temperature of 10 K and a strain rate of 1E10 /s. Prior to any loading application, minimization was performed using the conjugate gradient method. A cutoff distance of 9.5 Å was used for the Lennard–Jones and the long-range Coulombic pair potentials. To correctly account for charge screening and to ensure proper convergence of system energy due to contributions from long-range interactions, an Ewald summation with a desired relative error of $1\times 10^{-4}$ or less was used [29]. Due to the presence of hydrogen in the system, a time-step of 0.5 fs was used in all simulations. Validation of the interatomic potential choice and model size is consistent with prior work [21].

## 4. Conclusions

In this work, a molecular dynamics study was conducted to elucidate the effects of a spherical pore on the constitutive response and failure of periodic HAP single crystals subjected to uniaxial tension and compression along the X, Y, and Z directions. A size dependency of the mechanical response on pore diameter was discovered that varied based on the loading direction, with the Z axis being the least sensitive to the pore in tensile loading but the most sensitive, i.e., the most weakened, by the pore in compression. Such responses were related to the nanoscale deformation mechanisms uncovered for HAP single crystals in a previous study [21], with large pores disrupting the large numbers of X-Y planar ionic bonds formed from Ca polyhedra and thus inducing defect sensitivity in X and Y tension but not in Z, which is instead constitutively supported by large numbers of out of plane ionic and covalent bonds. However, differences in Coulombic repulsion induced by pores in compressive loading created a large degree of defect sensitivity in the Z direction—attributed to alterations of bond angle closure behavior, which drives compressive failure in Z but not in X or Y. While atomic shear strain concentrations at the circumferential edges of the pores indicate a significant contribution of pore-induced charge imbalance and geometric stress concentration in failure relative to pristine HAP, the aforementioned deformation mechanisms gleaned from pristine crystal studies likely play an important role and warrant further investigation. This work provides the basis for a deeper understanding of the mechanical response of bulk HAP containing defects commonly formed in manufacturing processes—exhibiting great potential for informing current and future processes and structure designs of synthetic HAP used in biomedical applications.

## Data Availability

Source data files are available from the corresponding author upon request and with approval from Northern Illinois University.

## Abbreviations

The following abbreviations are used in this manuscript:

HAP	Hydroxyapatite
MD	Molecular Dynamics
CVFF	Consistent Valence Force Field
